# Draft Genome Sequence of the Organophosphorus-Degrading Bacterium *Pseudomonas* sp. Strain 1-7, Isolated from Organophosphorus-Polluted Sludge

**DOI:** 10.1128/genomeA.00993-14

**Published:** 2014-10-02

**Authors:** Jian Tian, Li Xu, Shuangyu Zhang, Wen Sun, Xiaoyu Chu, Ningfeng Wu

**Affiliations:** a Biotechnology Research Institute, Chinese Academy of Agricultural Sciences, Beijing, China; b Environmental Protection Research Institute of Light Industry, Beijing, China

## Abstract

*Pseudomonas* sp. strain 1-7, isolated from organophosphorus-polluted sludge, is able to degrade many organophosphorus compounds. Here, we report the draft genome sequence of *Pseudomonas* sp. strain 1-7.

## GENOME ANNOUNCEMENT

Organophosphorus compounds (OPCs) belong to a class of highly toxic neurotoxins that are widely used as insecticides (1). The residues of such compounds in the environment have contaminated much soil and ground water in the world (2–4). The *Pseudomonas* sp. strain 1-7 was isolated from the organophosphorus-polluted sludge of a pesticide factory in Tianjin, China (5). The strain can degrade many OPCs, such as methyl parathion (MP), parathion, chlorpyrifos, and so on. In addition, the strain also can totally degrade the *para*-nitrophenol (PNP), a main hydrolysate of OPCs with two different pathways (5). Here, we report the draft genome sequence of *Pseudomonas* sp. strain 1-7.

Whole-genome sequencing was carried out using Illumina HiSeq 2000 technology with a 300-bp paired-end library and a 3-kb mate-pair library, which generated 8,069,344 high-quality (Phred score cutoff of 20) reads (approx. 120× coverage of the genome), and 11,277,836 high-quality reads (approx. 200× coverage of the genome), respectively. The reads were *de novo* assembled with the software SOAPdenovo version 2.01 (http://soap.genomics.org.cn/soapdenovo.html). As a result, the draft genome sequence of *Pseudomonas* sp. strain 1-7 comprises 4,929,379 bp, with an average GC content of 62.0%, consisting of 74 contigs (*N*_50_, 178,055 bp). Automatic gene annotation was carried out by the NCBI Prokaryotic Genomes Automatic Annotation Pipeline (PGAAP) (http://www.ncbi.nlm.nih.gov/genome/annotation_prok) and was followed by manual editing. The genome sequence contains 4,094 candidate protein-coding genes, giving a coding intensity of 74.2%, and the average size of each gene is 894 bp. In addition, 61 tRNA genes for 20 amino acids and 5 16S-23S-5SrRNA operons were identified in the genome.

The analysis of the genome data from *Pseudomonas* sp. strain 1-7 revealed that there was a gene of methyl parathion hydrolase (*mph*, accession number FJ821775) and a gene cluster (accession number JX854037.1) for degrading of PNP, the main hydrolysate of methyl parathion by MPH (5). Additionally, more than 30 genes related to degradation of the other organophosphorus and aromatic compounds were found, including atrazine, 1,1,1-trichloro-2,2-bis(4-chlorophenyl)ethane (DDT), gamma-Hexachlorocyclohexane, atrazine, toluene, and so on. The genome information and annotation reported in the present study are valuable for future research to investigate organophosphorus compound degradation in environments (6) and bioremediation of soil and water contaminated by organophosphorus compounds (7, 8).

### Nucleotide sequence accession numbers.

The whole-genome shotgun project for *Pseudomonas* sp. strain 1-7 has been deposited at DDBJ/EMBL/GenBank under the accession number JPRQ00000000. The version described in this paper is version JPRQ01000000.
